# Secretagogin Is Highly Expressed in Enteroendocrine K Cells and Plays a Critical Role in Nutrient-Induced GIP Secretion

**DOI:** 10.1210/jendso/bvaf022

**Published:** 2025-02-21

**Authors:** Xinyu Liu, Xuan Liu, Yuanyuan Hu, Xin Wang, Xin Yang, Bin Yan, Yiting Zhou, Lingzhi Zhou, Gang Fan, Jing Yang

**Affiliations:** Department of Endocrinology, The Sixth Affiliated Hospital of Shenzhen University Health Science Center, Shenzhen Nanshan People's Hospital, Shenzhen 518052, Guangdong, China; Department of Endocrinology, The First Affiliated Hospital, Hengyang Medical School, University of South China, Hengyang 421001, Hunan, China; Department of Endocrinology, The Central Hospital of Shaoyang City, Shaoyang 422000, Hunan, China; Department of Endocrinology, The Sixth Affiliated Hospital of Shenzhen University Health Science Center, Shenzhen Nanshan People's Hospital, Shenzhen 518052, Guangdong, China; Department of Endocrinology, The First Affiliated Hospital, Hengyang Medical School, University of South China, Hengyang 421001, Hunan, China; Department of Endocrinology, The First Affiliated Hospital, Hengyang Medical School, University of South China, Hengyang 421001, Hunan, China; Department of Endocrinology, The First Affiliated Hospital, Hengyang Medical School, University of South China, Hengyang 421001, Hunan, China; Department of Endocrinology, The First Affiliated Hospital, Hengyang Medical School, University of South China, Hengyang 421001, Hunan, China; Department of Pediatrics, The Sixth Affiliated Hospital of Shenzhen University Health Science Center, Shenzhen Nanshan People's Hospital, Shenzhen 518052, Guangdong, China; Department of Urology, The Sixth Affiliated Hospital of Shenzhen University Health Science Center, Shenzhen Nanshan People's Hospital, Shenzhen 518052, Guangdong, China; Department of Endocrinology, The Sixth Affiliated Hospital of Shenzhen University Health Science Center, Shenzhen Nanshan People's Hospital, Shenzhen 518052, Guangdong, China; Department of Endocrinology, The First Affiliated Hospital, Hengyang Medical School, University of South China, Hengyang 421001, Hunan, China

**Keywords:** secretagogin (SCGN), glucose-dependent insulinotropic polypeptide (GIP), K cell, type 2 diabetes mellitus (T2DM)

## Abstract

**Context:**

Incretin hormones, primarily composed of glucose-dependent insulinotropic polypeptide (GIP) and glucagon-like peptide-1 (GLP-1), are secreted by enteroendocrine cells (EECs) and play crucial roles in maintaining blood glucose homeostasis. Notably, GIP accounts for two-thirds of the entire incretin effect. However, the secretion and function of GIP are impaired in individuals with type 2 diabetes mellitus (T2DM), and the regulatory mechanisms governing GIP secretion remain unclear.

**Objective:**

Our study aims to explore the role of an EEC-enriched protein, Secretagogin (SCGN), in the regulation of GIP secretion.

**Methods:**

We collected duodenal tissues from both humans and mice to observe the colocalization of SCGN and GIP in EECs. Additionally, we utilized human cohorts and gene-edited mouse models to investigate the effect of SCGN on GIP secretion. Our study included 128 subjects, comprising 64 individuals diagnosed with newly onset diabetes and 64 age- and sex-matched nondiabetic healthy controls. At the animal level, we employed leptin receptor–deficient (db/db) mice and *Scgn* knockout mice for our investigations.

**Results:**

Our findings indicate that SCGN is abundantly expressed in GIP-producing K cells within the intestinal epithelium of both humans and mice. We observed a positive correlation between SCGN and GIP levels in postprandial states among patients with T2DM, db/db mice, and their healthy controls. Notably, *Scgn* knockout mice exhibited decreased GIP and insulin secretion. However, SCGN deficiency did not affect K-cell number, GIP mRNA expression, or intestinal morphology.

**Conclusion:**

Collectively, these findings demonstrate that SCGN is a key regulator of nutrient-induced GIP secretion.

Type 2 diabetes mellitus (T2DM) is a condition characterized by insulin resistance, inadequate pancreatic insulin secretion, and dysregulated glucagon secretion [1]. Glucose-dependent insulinotropic polypeptide (GIP), produced by K cells in the duodenum and jejunum, and glucagon-like peptide-1 (GLP-1), synthesized by L cells in the small intestine and colon, both play critical roles in the regulating glucose homeostasis [2]. GIP and GLP-1 collaborate to enhance postprandial insulin secretion, thereby facilitating the efficient disposal of glucose following a meal through a phenomenon known as the incretin effect [3]. This effect refers to the enhanced insulin response to oral glucose, primarily mediated by the actions of the incretin hormones GIP and GLP-1 on incretin receptors located on pancreatic β-cells. The presence of nutrients, particularly carbohydrates and fats, in the digestive system triggers the release of these hormones. Research has shown that in healthy individuals, the incretin effect accounts for approximately 70% of postprandial insulin secretion, with GIP contributing around 44% [4]. This makes GIP as the primary contributor to the incretin effect and a significant factor in postprandial glucose regulation [5].

Although GIP is the primary incretin hormone in healthy individuals and plays a crucial role in incretin effects, its insulin response is significantly reduced in individuals with T2DM who have uncontrolled hyperglycemia, a phenomenon referred to as GIP resistance [6]. This resistance is attributed to reduced GIP receptor expression and postreceptor defects associated with prolonged β cell stimulation [7]. As a result, GIP has traditionally not been considered a viable therapeutic target for T2DM. Intriguingly, recent studies have demonstrated that the insulinotropic effectiveness of GIP can be partially restored through improved glycemic control [8, 9]. These promising discoveries have paved the way for the development of therapies based on GIP receptor agonists or strategies aimed at enhancing the endogenous secretion of GIP for the treatment of T2DM [10].

In the regulation of incretin secretion, the soluble N-ethylmaleimide-sensitive factor attachment protein receptors (SNARE) complex plays a critical role [11]. Secretagogin (SCGN), an EF-hand calcium binding protein, is primarily found in islet cells, specific regions of the brain, and enteroendocrine cells (EECs). SCGN interacts with the core SNARE protein SNAP25 and β-actin, functioning as a key regulator of the vesicular secretion of hormones such as insulin, corticotropin-releasing hormone, and oxytocin, among others [10, 12-16]. Recent research has shown that SCGN is colocalized with GLP-1 in intestinal L-cells and plays a pivotal role in the circadian release of GLP-1 [17]. However, it remains unclear whether SCGN also regulates the secretion of GIP. Given the significant similarity in secretion mechanisms between GIP and GLP-1, we hypothesized that SCGN may have the potential to regulate GIP secretion. Therefore, the main objective of this study is to examine the influence of SCGN on GIP secretion using both human plasma samples and *Scgn* knockout mouse models.

## Materials and Methods

### Cohort of Study and Human Duodenal Tissue

The study encompassed 128 participants from The Sixth Affiliated Hospital of Shenzhen University Health Science Center spanning the years 2023 to 2024. These comprised 64 individuals with newly diagnosed diabetes and 64 nondiabetic healthy control individuals from the Department of Metabolism and Endocrinology. Specifically, the inclusion criteria for patients with newly onset diabetes in this study required that participants be aged between 18 and 65 years, have a duration of diabetes of less than 1 year, and meet the 1999 World Health Organization diagnostic criteria for diabetes. Participants were excluded if they had a history of chronic illnesses or had been taking medications that affect glucose metabolism within the past 6 months. Additionally, pregnant or lactating individuals as well as those unable or unwilling to provide informed consent were also excluded. The healthy control cohort consisted of individuals aged 18 to 65 years, who had no history of chronic disease or regular medication consumption. Individuals with diabetes were excluded based on their levels of fasting blood glucose, postprandial blood glucose, and glycated hemoglobin. The clinical characteristics of the patients and control individuals are summarized in Table 1. Human duodenal tissues were obtained from 2 donors who underwent pancreaticoduodenectomy at The Sixth Affiliated Hospital of Shenzhen University Health Science Center as a treatment for cholangiocarcinoma. This study received approval from the Ethics Committee of The Sixth Affiliated Hospital of Shenzhen University Health Science Center, as well as The First Affiliated Hospital of the University of South China. Informed consent was obtained from all participants.

**Table 1.** Clinical characteristics of T2DM and healthy controls

	Healthy control	T2DM	*P*
Age (years)	42.2 ± 11.65	43.5 ± 11.98	.544
Gender (F/M)	36/28	30/34	.742
BMI (kg/m^2^)	23.85 ± 2.90	24.94 ± 2.67	.575
Diabetic duration (years)	−	1.89 ± 1.36	−
HbA1c (%)	5.44 ± 0.29	9.31 ± 1.58	<.001
FBG	5.33 ± 0.30	9.49 ± 1.62	<.001

Abbreviations: BMI, body mass index; FBG, fasting blood glucose; T2DM, type 2 diabetes mellitus.

### Animals and Treatment

Seven-week-old male db/db (C57BL/6J) and wild-type (WT) male mice were purchased from Hunan BoRuiXin biological Technology Co. Ltd. Global *Scgn* knockout mice were created using the CRISPR/Cas9 system by Cyagen Biosciences Inc. (Guangzhou, China). Specifically, 11 exons are identified in *Scgn* gene exon (transcript: ENSMUST00000021770) and exon 2∼3 were selected as target site. The strategy for *Scgn* KO generation is shown in Fig. S1 [18]. Two single guide RNAs (sgRNA1 and sgRNA2) targeting S*cgn* exons 2 and 3 were designed. Cas9 mRNA and gRNA generated by in vitro transcription were then injected into the fertilized eggs for *Scgn* knockout mouse production. The pups were genotyped by polymerase chain reaction (PCR) followed by sequencing analysis. The PCR primers used for *Scgn* KO mice identification were listed in Table S1 [19].

All mice were housed in an air-controlled room at a temperature of 25 °C, with a dark–light cycle of 10 hours of darkness and 14 hours of light. They had free access to water and were provided with a normal chow diet food (3.73 kcal/g; 12% fat, 23% protein, and 65% carbohydrate; Department of Laboratory Animals, University of South China).

The above animal experimentations were conducted in accord with accepted standards of humane animal care, and approved by the Committee on Animal Care and Use of the Ethics Committee of The First Affiliated Hospital of University of South China.

### Oral Lard Oil Tolerance Test and Oral Glucose Tolerance Test (OGTT)

After a 12-hour fasting period, oral lard oil tolerance test (OLTT) was performed, administering 10 mL/kg of lard oil. Blood samples were collected via the tail vein at 0, 30, 60, and 90 minutes following oral oil administration through gavage. Plasma levels of insulin and GIP were measured using enzyme-linked immunosorbent assay (ELISA) kits as detailed below.

Similarly, an oral glucose tolerance test (OGTT) was also conducted to evaluate GIP secretion. A glucose solution (2 g/kg body weight) was prepared in sterile distilled water and administered to the mice via oral gavage. Blood samples were collected from the tail vein at 0, 30, 60, and 90 minutes following glucose administration. Plasma levels of insulin and GIP were measured.

### Immunofluorescence Staining

The immunofluorescence technique was utilized to examine the distribution and colocalization of SCGN and GIP proteins in duodenal K cells, as well as to assess the impact of *Scgn* knockout on the expression of SCCN in pancreatic islets. Initially, paraffin-embedded duodenal or pancreas tissues were sliced and fixed. The samples were then dewaxed, rehydrated, and blocked with 10% normal goat plasma for 2 hours. Following this, the tissues were incubated overnight with primary antibodies against SCGN (RRID:AB_10989370) and GIP (RRID:AB_2813903) at a dilution of 1:100. Afterward, the samples were incubated for 1 hour with CoraLite 488- and 594- labeled secondary antibodies (RRID:AB_2797132) (RRID:AB_2810984). Finally, the fluorescence signals were visualized using a fluorescence microscope (Axioscope 5; Zeiss, Oberkochen, Germany).

### Hematoxylin and Eosin Staining

The morphology of intestinal tissue cells was examined utilizing the standard hematoxylin and eosin staining technique. Initially, specimens from the duodenum, jejunum, and colon were fixed in 10% neutral buffered formalin for 24 hours, followed by dehydration and embedding in paraffin. The tissue sections were cut to a thickness of 5 μm. After deparaffinization and hydration, the sections were immersed in hematoxylin staining solution for approximately 3 minutes to stain the cell nuclei, followed by a brief rinse and transfer to eosin staining solution for about 1 minute to stain the cytoplasm. Subsequently, the sections underwent dehydration through increasing concentrations of alcohol. After dehydration, the sections were cleared and mounted with DPX mounting medium. The stained sections were then observed under an optical microscope, and images were captured and analyzed using an image analysis system.

For intestinal morphology measurement, the length from the tip of the villus to the orifice of the crypt is defined as villus length, while the length from the orifice of the crypt to the base of the crypt is defined as crypt length. The length of the median axis perpendicular to the crypt and villi is referred to as crypt width and villus width. The lengths and widths of villi, as well as the crypt lengths and crypt widths, were measured to compare the intestinal morphology among WT, *Scgn* heterozygote deficient (*Scgn*^+/−^), and *Scgn* knockout (*Scgn*^−/−^) mice.

### Enzyme-Linked Immunosorbent Assay

Plasma insulin, GIP, and SCGN levels were measured using an ELISA kit according to the provided instructions. Human and mouse insulin ELISA kits were obtained from American Laboratory Products Company (ALPCO), NH, USA (RRID:AB_2801438) (RRID:AB_3674340). Additionally, human and mouse GIP ELISA kits were sourced from Merck Millipore, Billerica, MA (RRID:AB_2801401) (RRID:AB_2801384), while human and mouse SCGN ELISA kits were procured from MyBioSource, CA, USA (RRID:AB_3674343) (RRID:AB_3674344).

### Reverse Transcription Quantitative Polymerase Chain Reaction

Intestinal samples used for reverse transcription (RT) quantitative (q)PCR assay were collected from the segment extending from the pylorus to the ligament of Treitz. Total RNA was subsequently extracted using TRIzol reagent (Thermo Fisher Scientific, Inc.) and reverse transcribed with MMLV reverse transcription reagents at 37 °C for 1 hour (Promega Corporation). Relative qPCR was performed using SYBR-Green detection chemistry (Qiagen, Inc.) on a PRISM 7500HT real-time PCR system (Applied Biosystems; Thermo Fisher Scientific, Inc.). The thermocycling reaction consisted of the following conditions: one cycle at 95 °C for 15 minutes, followed by 38 cycles of denaturation at 95 °C for 15 seconds, annealing at 60 °C for 15 seconds, and extension at 70 °C for 10 seconds. The expression level of *GIP* and *Scgn* mRNA was validated using RT-qPCR, with *Gapdh* serving as the endogenous control. The relative gene expression levels were analyzed using the 2^−ΔΔCq^ method. The primers used for PCR amplification are detailed in Table S1 [19].

### Statistical Analysis

In this population-based study, we conducted statistical analyses using SPSS version 20.0 software. Prior to the analyses, we employed propensity score matching to control for confounding variables, including age, gender, and body mass index. Normality tests and chi-square tests were performed before making comparisons. If the assumptions of normality and equal variance were satisfied, we conducted an analysis of variance between the 2 groups using the independent samples t-test. Differences among multiple groups were analyzed using analysis of variance. Subsequently, Pearson correlation analysis was performed on data that adhered to a normal distribution, while the Mann–Whitney U test and Spearman correlation analysis were applied to data that did not follow a normal distribution. All tests were conducted as 2-tailed tests with a significance level set at *P* < .05.

## Results

### Colocalization of SCGN and GIP Was Observed in Both Male Mouse and Human Duodenal EECs.

**Figure 1.**
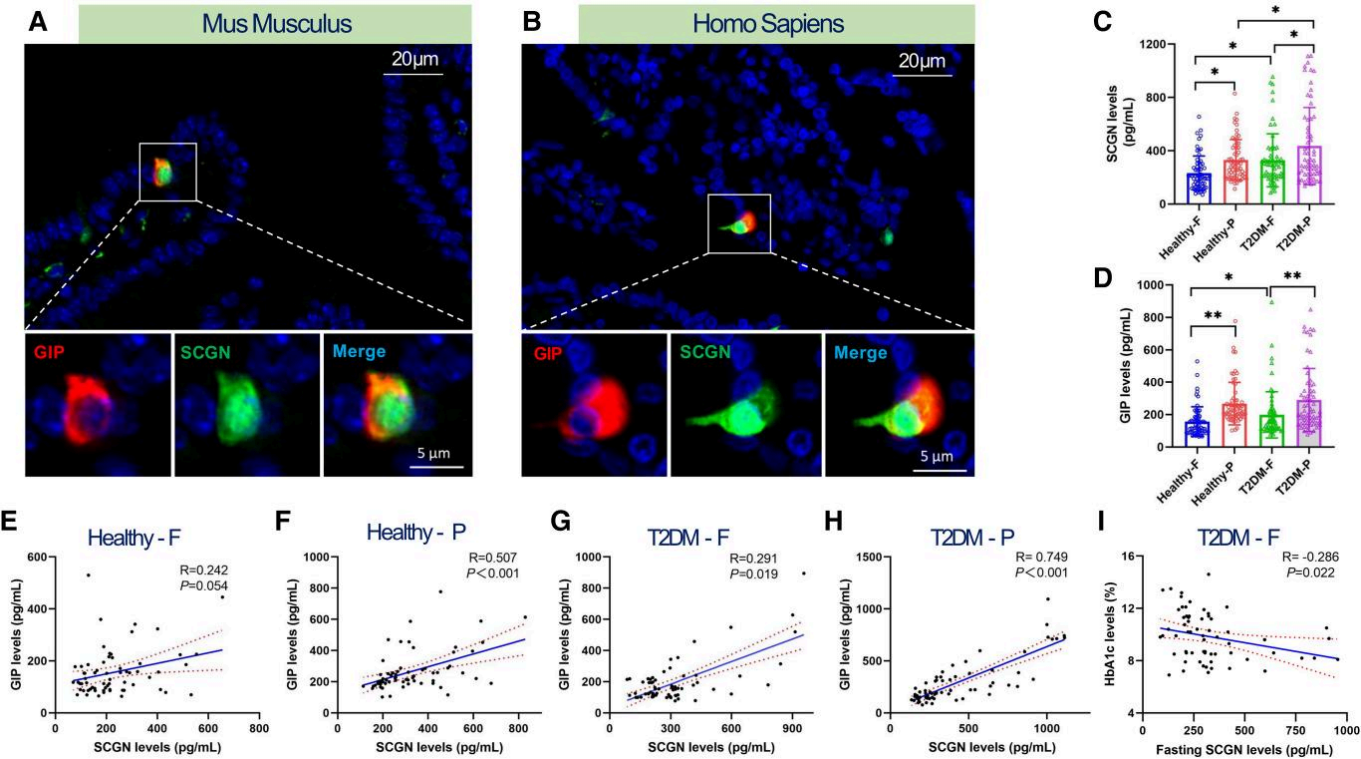
Coexpression of SCGN and GIP in EECs and the correlation of serum SCGN levels with GIP and HbA1c in healthy individuals and patients with T2DM. (A, B) Immunofluorescence colocalization assay showed that GIP is primarily localized in the cytoplasm, whereas SCGN is present in both the cytoplasm and the nucleus. Both proteins exhibited intense fluorescence signals concentrated in the cytoplasmic compartment of EECs. Scale bars are 20 μm for the upper images and 5 μm for the enlarged images. (C) Fasting and postprandial SCGN levels were measured in healthy individuals and patients with T2DM. F (fasting) refers to the period during which individuals refrain from eating from the previous evening's dinner until the following morning. In contrast, P (postprandial) state represents the condition occurring 2 hours after breakfast. (D) Fasting and postprandial GIP levels were assessed in the same populations. (E-H) The correlational relationship between fasting and postprandial SCGN and GIP levels in both healthy individuals and T2DM patients. (I) Correlation analysis of fasting SCGN and HbA1c levels in T2DM patients.

In our immunofluorescence colocalization study of human and mouse duodenal tissue samples, we observed that GIP is primarily localized in the cytoplasm, whereas SCGN is present in both the cytoplasm and the nucleus. Both proteins exhibited intense fluorescence signals concentrated in the cytoplasmic compartment of EECs, with minimal background staining. Merged images clearly demonstrated a high degree of colocalization, with SCGN and GIP signals predominantly overlapping within the cytoplasm (Fig. 1A). Similar results were also observed in human EECs (Fig. 1B).

### The Plasma Levels of SCGN Are Positively Correlated With GIP in Nondiabetic Individuals and Patients With Diabetes

We measured plasma levels of SCGN and GIP in both fasting and postprandial states among nondiabetic healthy individuals and patients with type 2 diabetes. The results indicated that SCGN and GIP levels increased postprandially in both groups, with a particularly pronounced rise noted in patients with type 2 diabetes (Fig. 1C and 1D). Subsequent correlation analysis revealed a significant association between postprandial SCGN and GIP levels in healthy individuals (R = 0.507; *P* < .01) (Fig. 1F), which is consistent with findings in patients with type 2 diabetes, showing correlations in both fasting (R = 0.291; *P* = .019) (Fig. 1G) and postprandial (R = 0.749; *P* < .001) (Fig. 1H) measurements. However, no significant correlation between plasma SCGN and GIP levels was observed in fasting healthy individuals (Fig. 1E). We also analyzed the relationship between fasting SCGN levels and HbA1c levels in patients with type 2 diabetes. Our findings are consistent with previous reports [20], revealing a negative correlation between the 2 variables (R = −0.286; *P* = .022) (Fig. 1I).

### The Plasma Levels of SCGN Are Positively Correlated With GIP in Male Mice

**Figure 2.**
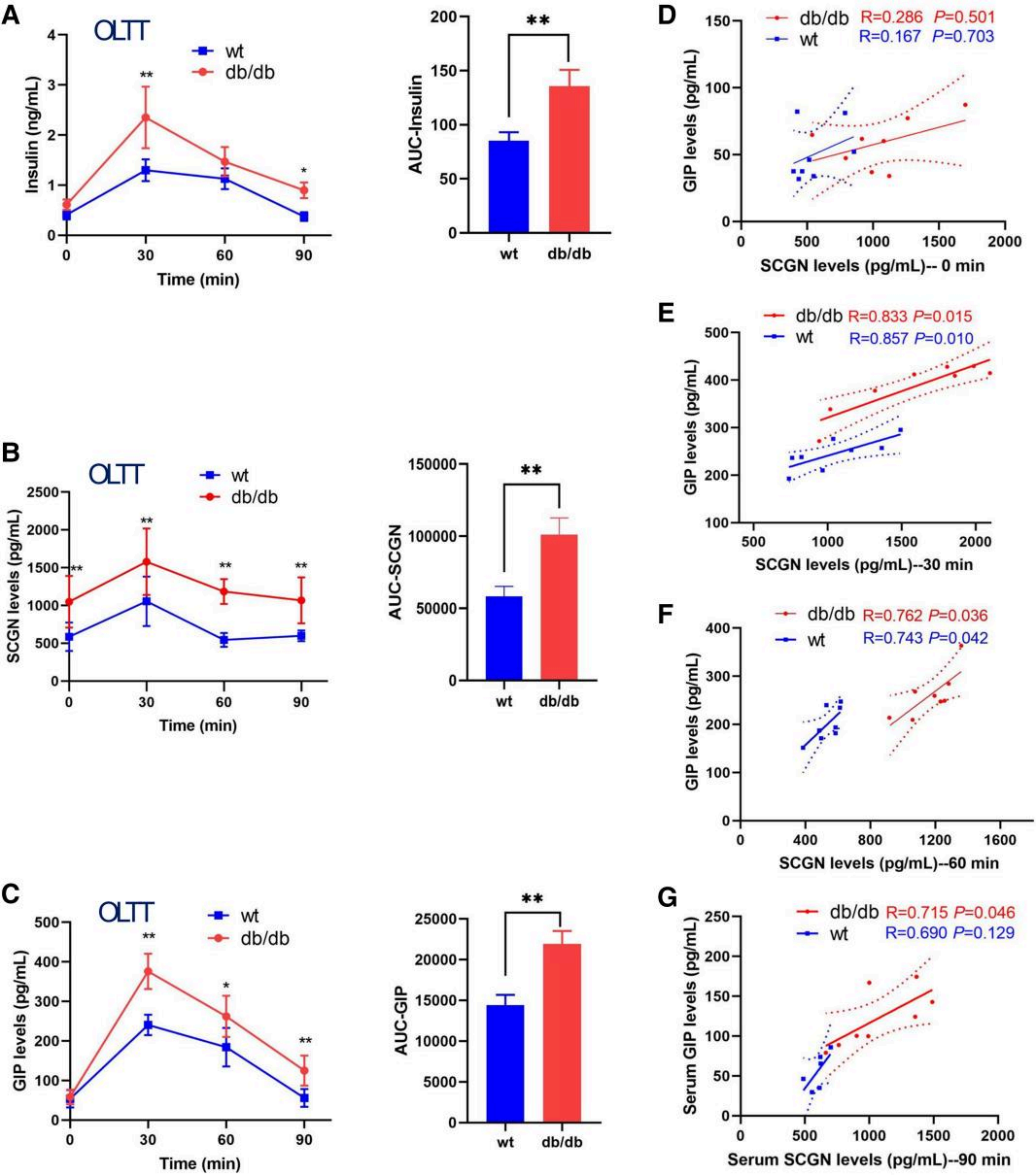
The plasma levels of SCGN are positively correlated with GIP in male mice. Fluctuations in plasma insulin levels (A), SCGN levels (B), and GIP levels (C) were measured at 0, 30, 60, and 90 minutes following the administration of lard oil in both db/db and WT mice. (D-G) Correlation analyses were conducted to examine the relationships between SCGN and GIP levels in both groups of mice at the 0-, 30-, 60-, and 90-minute time points.

To further clarify the relationship between SCGN and GIP, we conducted animal experiments involving 16 8-week-old male mice, which were divided into 2 groups: the diabetic group (db/db, n = 8) and the WT group (n = 8). Fluctuations in blood glucose levels, plasma insulin levels, SCGN levels, and GIP levels were recorded at 0, 30, 60, and 90 minutes following lard oil administration. Intriguingly, within 90 minutes postprandially, the db/db group exhibited significantly higher levels of insulin, SCGN, and GIP than the WT group (Fig. 2A-2C).

We subsequently conducted correlation analyses on plasma GIP levels and plasma SCGN levels in both groups. The results revealed significant correlations between plasma SCGN levels and plasma GIP levels in the db/db group mice at 30 minutes (R = 0.833, *P* = .015), 60 minutes (R = 0.762, *P* = .036), and 90 minutes (R = 0.715, *P* = .046) postprandially. Similarly, a significant correlation was observed at 30 and 60 minutes postprandially in the WT group mice (R = 0.857, *P* = .010 and R = 0.743, *P* = .042, respectively; see Fig. 2D-2G)

### *Scgn* Knockout Male Mice Show Impaired Nutrient-Induced Insulin and GIP Secretion

**Figure 3.**
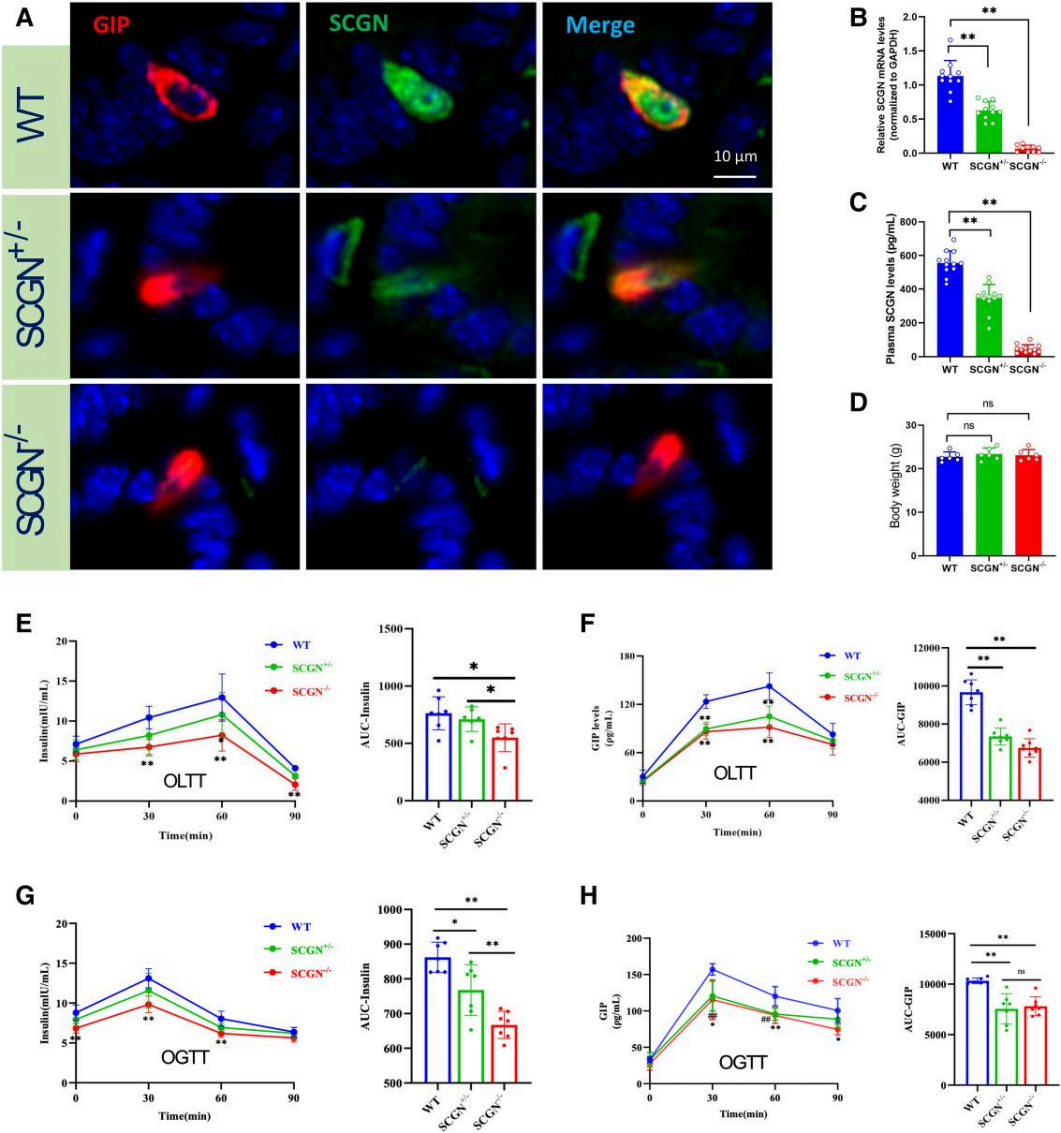
*Scgn* knockout impairs nutrient-induced insulin and GIP secretion in male mice. (A, B) The levels of SCGN protein and mRNA in EECs from *Scgn*  ^−/−^, *Scgn*  ^+/−^, and WT mice were assessed using immunofluorescence staining and qRT-PCR, respectively. (C) Plasma SCGN levels in the 3 groups of mice were quantified using an ELISA. (D) Body weight was measured among the 3 groups of 8-week-old mice. (E, F) The detailed levels and calculated area under the curve (AUC) for insulin, and GIP levels in response to OLTT in *Scgn*^−/−^ and *Scgn*^+/−^ mice compared with WT mice. (G, H) The changes of GIP and insulin levels in 3 groups of mice in response to OGTT.

We further investigated the influence of SCGN on GIP secretion in *Scgn* knockout mice, specifically 35 8-week-old C57BL/6J mice, which were divided into 3 groups: WT (n = 12), *Scgn* heterozygous knockout (*Scgn*^+/−^, n = 12), and *Scgn* homozygous knockout (*Scgn*^−/−^, n = 11). Initially, mice were genotyped using a combination of 3 short-range PCR assays (Fig. S2A) [21]. Subsequently, SCGN mRNA levels and protein levels were measured in intestinal tissues, while protein levels were also assessed in pancreatic tissues and plasma. Intestinal and pancreatic samples used for qRT-PCR and immunofluorescence studies were collected from mice following an OLTT. Immunofluorescence staining revealed a significant reduction in SCGN protein levels in duodenal EECs (Fig. 3A) and in the islets (Fig. S2B) [21] of *Scgn* knockout mice. Consistently, qRT-PCR results indicated that WT mice exhibited the highest levels of *Scgn* mRNA, whereas *Scgn*^−/−^ mice showed the lowest levels in the intestinal tract (Fig. 3B). Similarly, plasma SCGN levels were significantly decreased in *Scgn*^−/−^ mice (Fig. 3C). Additionally, there were no significant differences in body weight among these groups of mice when fed a normal diet (Fig. 3D).

OLTT experiments showed the postprandial insulin and GIP secretion levels increased rapidly, peaking at 60 minutes postprandial in WT mice. In contrast, *Scgn*^−/−^ and *Scgn*^+/−^ mice exhibited a slower increase and lower levels of these hormones(Fig. 3E and 3F). Similarly, OGTT results also demonstrated impaired GIP and insulin secretion in *Scgn*^−/−^ and *Scgn*^+/−^ mice compared with WT mice(Fig. 3G and 3H). These results substantiate that *Scgn* deficiency can reduce nutrient-induced insulin and GIP secretion.

### *Scgn* Knockout Did Not Influence the Number of K Cells or the Expression of GIP mRNA in the Duodenum

**Figure 4.**
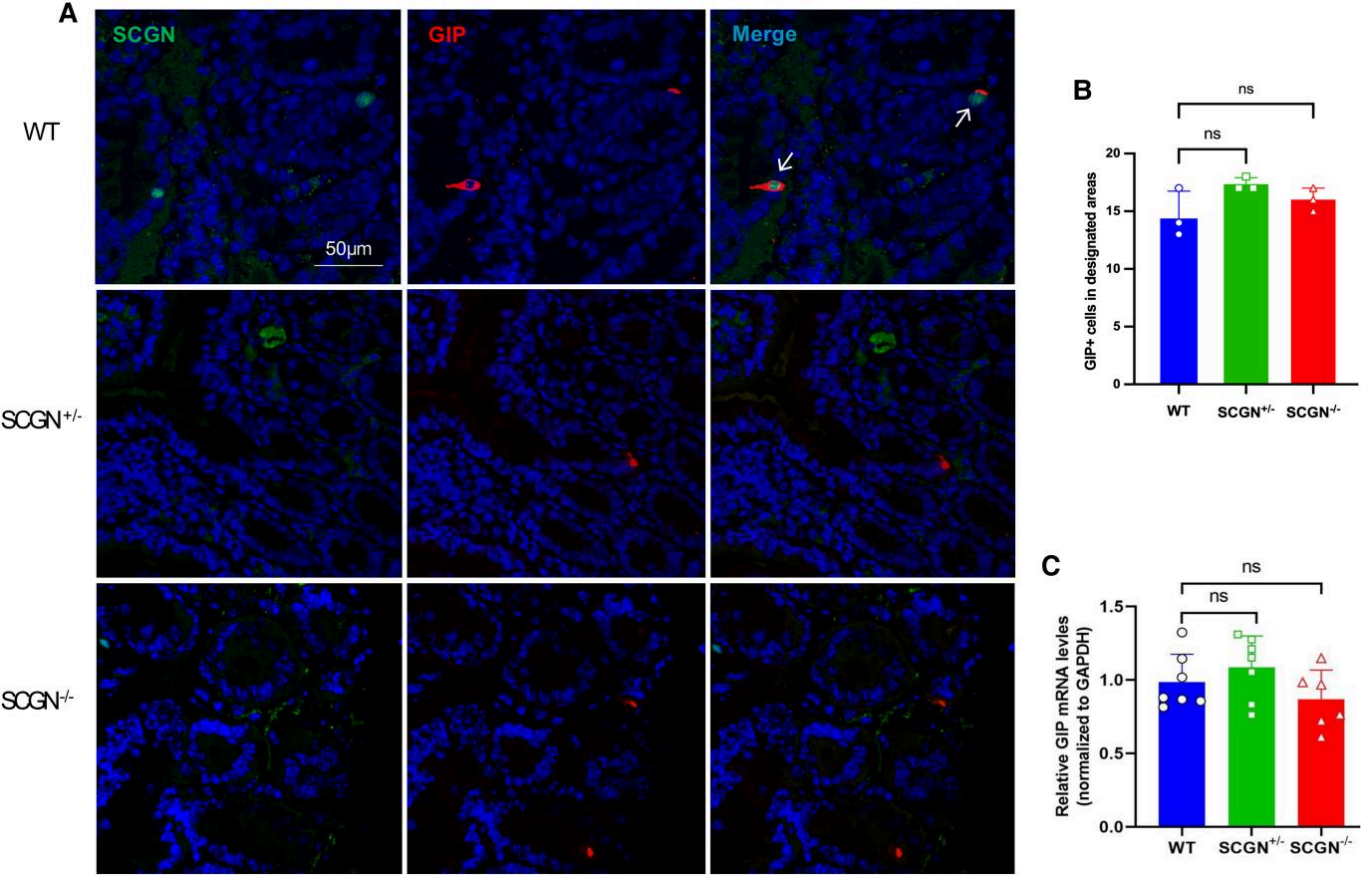
The influence of SCGN on the number of K cells and the expression of GIP mRNA in the duodenum. (A, B) The quantity of GIP positive cells in *Scgn*^−/−^, *Scgn*^+/−^ and WT mice was measured. (C) GIP mRNA levels in the duodenum among the 3 groups was assessed using qRT-PCR.

To determine whether *Scgn* knockout affects the number of K cells and GIP mRNA expression, we further calculated the number of K cells and the mRNA level in the upper small intestine. GIP-positive cells were identified as K cells, and the number of K cells in the duodenal area of 1 000 000 µm² was calculated for each mouse, with a total of 3 mice enrolled in each group. No significant differences in K cell numbers were observed among *Scgn*  ^−/−^, *Scgn*  ^+/−^, and WT mice (Fig. 4A and 4B). Additionally, there was no significant difference in GIP mRNA expression in the duodenum across the 3 groups (Fig. 4C). These findings suggest that *Scgn* deficiency does not impact the quantity of K cells or the synthesis of GIP mRNA.

### *Scgn* Knockout Did Not Affect Intestinal Morphology

**Figure 5.**
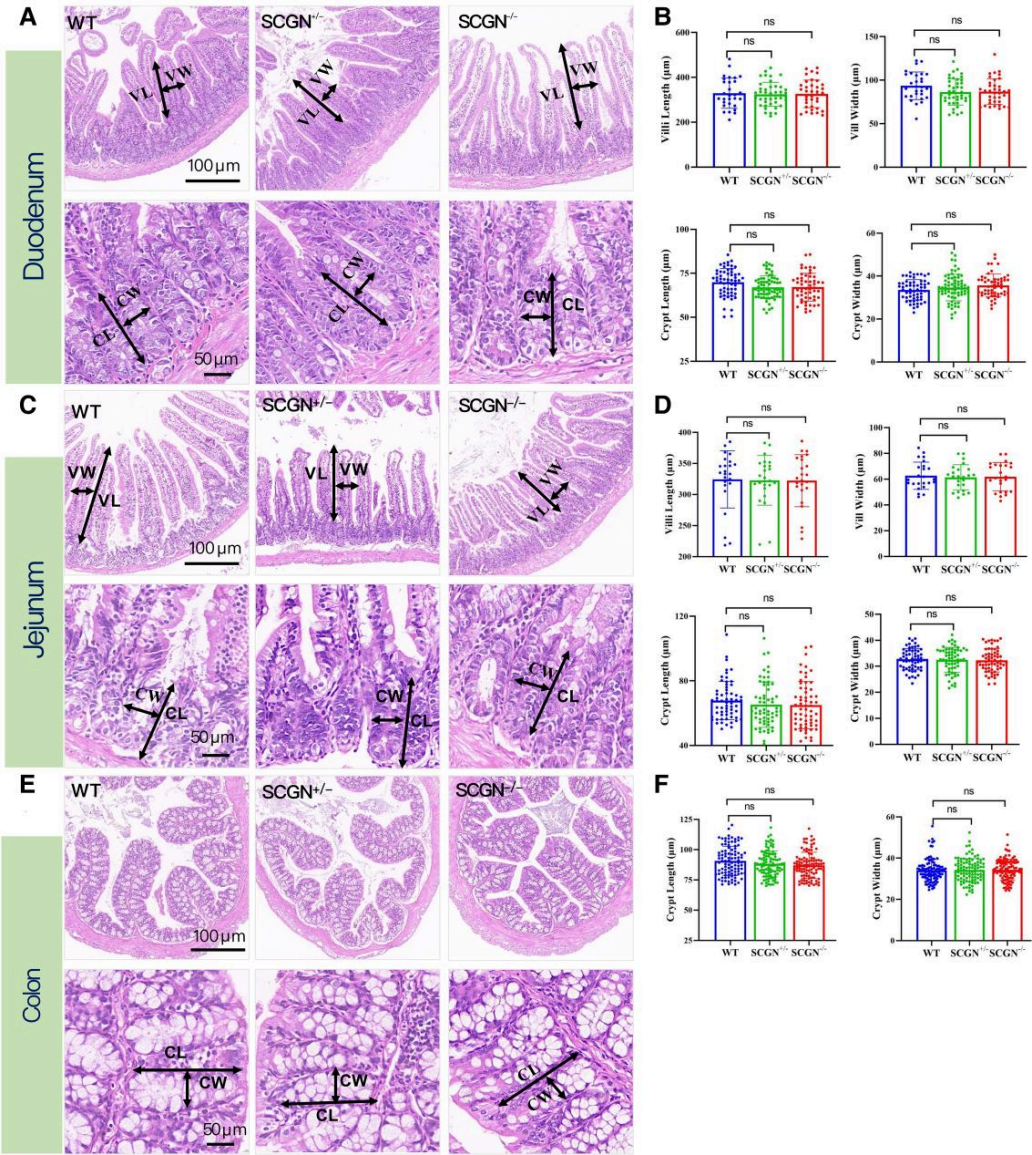
The impact of SCGN on the intestinal morphology. The impact of SCGN on intestinal morphology was assessed by examining villus length (VL), villus width (VW), crypt length (CL), and crypt width (CW) across 3 groups of mice in the intestinal segments of the duodenum (A, B), jejunum (C, D), and colon tissues (E, F).

Following hematoxylin and eosin staining of the duodenum, jejunum, and colon tissues from the 2 groups of gene-edited mice and WT mice, we evaluated the potential impact of SCGN on intestinal morphology in 20-week-old male mice. For each mouse, we measured 15 to 25 separate data points representing the characteristics of intestinal morphology in each intestinal segment, including villus length, villus width, crypt depth, and crypt width. Consequently, for each group of 3 mice, we obtained a total of 45 to 75 separate data points. These data were then statistically analyzed to provide a comprehensive assessment of intestinal morphology. Our analysis revealed no significant differences in villus length, villus width, crypt depth, or crypt width among the 3 groups in the intestinal segments examined (Fig. 5A-5F). These results suggest that *Scgn* deficiency had no significant effect on general intestinal morphology.

## Discussion

GLP-1 and GIP are incretin hormones secreted by EECs in the intestinal epithelial lining in response to food intake [3, 22]. These hormones play critical roles in managing glucose homeostasis and regulating appetite through both endocrine and neural mechanisms [23-25]. In these 2 hormones, GIP contributes approximately two-thirds of the entire incretin effect in insulin section. In patients with T2DM, the overall incretin effect is reduced [2, 26, 27]. The cellular mechanisms and intestinal cues that regulate endogenous GIP secretion continue to be areas of active research yet remain poorly understood.

In this study, we demonstrated that the calcium-binding protein SCGN is abundantly expressed in GIP-producing K cells within the human and mouse intestinal epithelium. The presence of SCGN in K cells, along with its colocalization with GIP, prompted us to investigate its previously unrecognized physiological roles in regulating GIP secretion. We observed a positive correlation between SCGN and GIP levels in postprandial states among patients with T2DM, leptin receptor gene knockout–induced diabetic mice (db/db mice), and their healthy controls. Consequently, we generated *Scgn* KO mouse models to further evaluate the function of SCGN in regulating GIP secretion. Our findings indicate that *Scgn* KO mice exhibit impaired tolerance to lard oil and glucose, characterized by decreased postprandial levels of GIP and insulin, alongside elevated triglyceride and glucose levels. Finally, we assessed K-cell numbers, GIP mRNA expression, and intestinal morphology in *Scgn* KO mice. Our results revealed that *Scgn* deficiency did not affect K-cell number, GIP mRNA expression, or intestinal morphology.

Previous studies have found that *Scgn* is expressed in intestinal L cells and plays a role in GLP-1 secretion. *Scgn* knockout mice and siRNA-treated cells exhibited significantly reduced GLP-1 secretion in response to nutrient stimulation [17]. SCGN was found to interact with SNAP25 and β-actin, which are components of the SNARE secretory complex, thereby regulating secretory granule dynamics [15, 28]. Meanwhile, the expression of SCGN is regulated by the clock gene *Bmal1*, leading to the circadian release of GLP-1 [17]. Similar to its role in GLP-1 secretion, our study revealed a positive correlation between SCGN and GIP in both patients with T2DM and healthy controls postprandially. We further verified the key role of SCGN in GIP secretion using knockout mice. Consequently, our study enhances the understanding of SCGN's function in regulating the secretion of incretin hormones in intestinal endocrine cells.

To clarify that the decrease in GIP secretion resulting from *Scgn* KO is not due to a reduction in K-cell number or GIP protein synthesis, we observed that *Scgn* KO has no significant effects on intestinal structure or the number of K cells, nor does it impact the synthesis of GIP mRNA. By analyzing the role of SCGN in regulating the secretion of GLP-1 and insulin, it is reasonable to postulate that SCGN regulates GIP secretion by influencing the process of vesicle secretion.

Given that SCGN can regulate the secretion of both GIP and GLP-1, key factors in facilitating weight loss [29], it is plausible to speculate that a decrease in SCGN levels may impact body weight. Although the current study found that *Scgn* gene knockout had no significant effect on the body weight of mice under short-term, normal-diet feeding, further investigation under long-term, nutrient-rich conditions will be crucial to elucidate this issue. Additionally, examining SCGN levels and exploring their relationship with GIP and GLP-1 in obese cohorts would be beneficial for validating this correlation.

Although this study established a well-defined relationship between SCGN and GIP secretion, several limitations should be acknowledged. Firstly, the precise mechanism by which SCGN regulates GIP secretion remains unclear, necessitating further investigation to determine whether the expression of SCGN and GIP secretion is also influenced by circadian proteins. Additionally, it is important to assess whether SCGN-regulated, stage-wise SNARE assembly plays a critical role in this process [14]. Secondly, the use of general *Scgn* knockout mice in this study complicates the interpretation of our findings, making it challenging to ascertain whether the observed decrease in insulin levels was a result of diminished GIP secretion or a direct consequence of SCGN's effect on insulin secretion. To address this, the use of K cell conditional knockout mice is necessary. Thirdly, the sample size in our clinical study is relatively limited, necessitating validation in a larger cohort in the future. Finally, another potential limitation is the possibility that *Scgn* deletion may also affect the release of other hormones with intestinotrophic effects, which could subsequently influence intestinal morphology and function. Future studies should investigate this aspect to enhance our understanding of the broader implications of SCGN deficiency.

Collectively, our findings demonstrate that SCGN is abundantly expressed in both human and mouse intestinal K cells, and that SCGN regulates nutrient-induced GIP secretion. *Scgn* deficiency leads to insufficient secretion of GIP and insulin, which disrupts the regulation of postprandial glucose levels. This disruption may subsequently elevate the risk of developing diabetes. By restoring intestinal SCGN expression and enhancing the secretion of incretin hormones, we anticipate the potential for new therapeutic strategies in the treatment of diabetes.

## Data Availability

The data supporting this study will be available from the corresponding author upon reasonable request.

## Abbreviations

EEC: enteroendocrine cell
ELISA: enzyme-linked immunosorbent assay
GIP: glucose-dependent insulinotropic polypeptide
GLP-1: glucagon-like peptide-1
OGTT: oral glucose tolerance test
OLTT: oral lard oil tolerance test
qPCR: quantitative polymerase chain reaction
RT: reverse transcription
SCGN: secretagogin
SNARE: soluble N-ethylmaleimide-sensitive factor attachment protein receptors
T2DM: type 2 diabetes mellitus
WT: wild-type
